# Three-dimensional reconstruction of ultrasound imaging in dentistry: a systematic review

**DOI:** 10.1007/s00784-026-07243-6

**Published:** 2026-11-05

**Authors:** Andrew Nalley, Rosa Emilia Gómez Torres, Kuo Feng Hung, Ray Tanaka, Takafumi Hayashi, Mihai Tarce

**Affiliations:** 1 Oral and Maxillofacial Radiology, Applied Oral Sciences and Community Dental Care, Faculty of Dentistry, The University of Hong Kong, Hong Kong, China; 2 Department of Periodontology, Institución Universitaria Colegios de Colombia (UNICOC), Cali, Colombia; 3 Division of Oral and Maxillofacial Radiology, Niigata University Graduate School of Medicine, Dentistry and Health Sciences, Niigata, Japan; 4 Periodontology and Implant Dentistry, Faculty of Dentistry, The University of Hong Kong, Hong Kong, China

**Keywords:** Dental ultrasonography, Three-dimensional ultrasound, Volumetric reconstruction, Intraoral ultrasound

## Abstract

**Background:**

Three-dimensional ultrasound (3D-US) reconstruction has emerged as a potential solution to the limited field of view and operator dependence of two-dimensional intraoral ultrasonography. Multiple acquisition, tracking, and reconstruction strategies have been proposed, yet no prior systematic review has synthesized the methods used specifically within dentistry.

**Objective:**

To identify and compare 3D-US reconstruction techniques applied in dentistry and evaluate how acquisition strategy, spatial tracking, and reconstruction methodology influence accuracy, reproducibility, feasibility, and clinical applicability.

**Methods:**

A systematic search of 5 databases was performed. Studies were included in the review if they used ultrasound imaging with any 3D-US reconstruction method for a defined dental application. Risk of bias was assessed using a customized hybrid QUADAS-2/QUIN/STARD framework.

**Results:**

Thirteen studies (2010–2025) met the inclusion criteria; 10 were in vitro and 3 in vivo. Acquisition methods included mechanized linear-stage scanning (*n* = 6), robotic arm control (*n* = 2), tracked freehand (*n* = 2), untracked freehand (*n* = 1), and custom intraoral jigs (*n* = 2). Accuracy ranged from 12 to 50 μm for digital impressions to sub-millimeter deviations for periodontal and ridge assessments, with most error rates below 10%. Reproducibility and clinical feasibility were both inversely related to hardware complexity: mechanized and robotic systems achieved the highest precision and enabled precise positioning for repeated evaluations; however, they had the lowest clinical usability. Conversely, freehand approaches demonstrated the strongest clinical potential but at the expense of reproducible positioning and highly precise measurements. Risk of bias was high or unclear in all studies due to heterogeneity, small sample sizes, and limited in vivo validation.

**Conclusions:**

Over 15 years, 3D-US reconstruction in dentistry has advanced substantially, demonstrating promising accuracy for digital impressions, periodontal assessment, ridge measurement, and implant planning. However, most studies remain feasibility-based and in vitro, and methodological variation limits comparability. Future progress will depend on standardization of validation frameworks, increased in vivo accuracy studies, and development of compact, automated acquisition workflows. With continued refinement, 3D-US has the potential to become a clinically practical, radiation-free imaging modality for diagnostic and procedural applications in dentistry.

**Supplementary Information:**

The online version contains supplementary material available at https://doi.org/10.1007/s00784-026-07243-6.

## Background

Diagnostic ultrasonography (US) provides a real-time, non-invasive, non-ionizing, accessible image with multiple clinical applications [1, 2]. The earliest use of US for dental purposes has been reported to be in 1963 when an ophthalmological scanner was used to view the internal structure of teeth [3]. Since then, the practical and potential applications of US have expanded into multiple domains. In the dental and maxillofacial area, applications include imaging of thyroid and salivary glands, evaluation of musculature tonicity and thickness, screening for lymph node metastases, evaluation of the temporomandibular joint and disc status, investigation into oral cancer including tongue carcinoma, evaluation of periodontal bone loss and furcation involvement, visualization of cortical bone erosion around dental periapical lesions, peri-implant visualization, and assessment of teeth integrity [2, 4–10].

Despite these advantages, conventional US remains limited by a restricted field of view, inability to image through mineralized tissues, and dependence on operator skill to capture the region of interest. In the dental environment, these limitations are further compounded by the confined intraoral environment, restricted probe maneuverability and angulation, and frequent acoustic shadowing from enamel and bone [4, 5].

3D-US reconstruction from sequential two-dimensional images has been explored as a strategy to extend spatial context and improve anatomical representation. This method was first introduced in 1986 for use in fetal imaging [11]. This three-dimensional ultrasound (3D-US) system introduces additional complexity into US imaging, but has also found a range of uses in medicine.

A range of technical strategies have been proposed to enable 3D-US reconstruction in dental applications. The approaches for creating these images vary with respect to probe motion control, spatial location, and reconstruction algorithms. Systems differ in their reliance on freehand scanning, mechanized or robotic positioning, external pose tracking or custom intraoral fixtures to establish spatial correspondence between sequential US images. This methodological heterogeneity reflects the absence of a universally accepted framework for dental 3D-US reconstruction and complicates comparison across studies.

While prior systematic reviews have explored freehand 3D-US techniques in broader medical contexts [12], these findings are not directly transferable to dentistry due to differences in anatomical access, imaging depth, and intraoral constraints. In contrast, existing dental systematic reviews have focused primarily on conventional two-dimensional US techniques and dental applications [8, 9, 13–18]. Collectively, these reviews identified US as a promising imaging modality for evaluating soft tissue thickness, periodontal structures, peri-implant tissues, jaw lesions, and cortical and crestal bone levels, often demonstrating agreement with direct measurements, histologic findings, CBCT, and cephalometric imaging. Commonly cited advantages include the absence of ionizing radiation, real-time image acquisition, and the ability of advanced Doppler techniques to assess vascularity and tissue perfusion. However, these reviews also consistently reported limitations related to methodological heterogeneity, variation in hardware and imaging protocols, small sample sizes, anatomical restrictions, operator dependency and training requirements, lack of dedicated intraoral transducers, limited integration into digital workflows, and the absence of standardized imaging approaches. Importantly, several reviews highlighted the need for dedicated intraoral hardware, standardized imaging protocols, and improved integration with digital workflows [8, 13, 17, 18], while 3D-US was identified as an important future direction [9]. However, none specifically evaluated the acquisition strategies, tracking approaches, and reconstruction methodologies used to generate 3D-US volumes.

Therefore, this systematic review aimed to synthesize the available evidence on 3D-US in dentistry, with particular emphasis on acquisition strategies, tracking methodologies, reconstruction approaches, and their implications for clinical implementation.

## Materials and methods

This systematic review was reported following the Preferred Reporting Items for Systematic Reviews and Meta-Analyses (PRISMA) [19] guidelines. The study protocol was developed prior to study selection and data extraction and was subsequently registered with the International Prospective Register of Systematic Reviews, PROSPERO (registration code: CRD420251110618). The initial literature search was conducted in June 2025, whereas the PROSPERO registration was finalized in September 2025. No substantive changes were made to the eligibility criteria, outcomes, or search strategy between protocol development and registration.

### Research question

The primary research question was: “What methods have been used to reconstruct 3D-US images in dentistry, and how do differences in acquisition strategy, spatial tracking and reconstruction approach affect measurement reliability and clinical applicability?”

The corresponding PICO framework was defined as:

- Population (P): Dental and maxillofacial imaging applications;
- Intervention (I): 3D-US reconstruction using varying acquisition and tracking strategies;
- Comparison (C): Alternative reconstruction methods or methodological approaches;
- Outcome (O): Accuracy, reproducibility, feasibility, and clinical applicability.

For the purposes of this review, “three-dimensional ultrasound” (3D-US) refers to any imaging output generated from US data that provides a three-dimensional (3D) respresentation through reconstruction of sequential 2D US acquisitions into a 3D dataset or model.

### Article identification

A search strategy was developed that aimed to identify articles from 3 main concept groups, each combined with the Boolean operator OR. Group 1 sought terms related to ultrasound imaging, group 2 comprised a 3-dimensional model constructed from the US images, while group 3 specified applications in dentistry. The groups were then combined with the Boolean operator AND. In June, 2025, a search was conducted into five electronic databases: PubMed, Embase, Cochrane Library, Web of Science and Medline. The search terms were modified for each database. The search was conducted to identify articles up to February 2025. An updated search was performed in March, 2026, to capture studies between March and December 2025.

The specific search terms for each database are displayed in Table S1.

The study selection process was completed in two stages by two independent reviewers (A.N.; R.E.G.T.). In the first stage, the title and abstract were reviewed. If the title and abstract contained information that was likely relevant to a 3D-US model with a dental application, the article was retained for full-text assessment. In cases that were unclear from title and abstract review, the full-text was retained. In the second stage, the full texts of selected articles were retrieved and assessed by the same reviewers compared to the inclusion and exclusion criteria. A third reviewer (M.T.) was available for consensus for any articles that were unclear for inclusion.

The inclusion criteria for this review were peer-reviewed original articles for initial, pilot, validation or clinical studies that utilized US imaging and different techniques of 3D-US reconstruction to create a 3D model with specified applications in dentistry.

The exclusion criteria for this review included:

- Articles not published in English;
- Articles that did not utilize US imaging techniques;
- Articles that did not combine US imaging into a 3D volume;
- Articles that do not indicate potential clinical applications in dentistry.

### Data extraction

Data extraction was conducted independently by the same reviewers (A.N.; R.E.G.T.). A template was created in an Excel spreadsheet (Microsoft Corporation, Redmond, Washington, USA). The following data items were extracted from each article:

1. If the study was conducted in an in vivo or in vitro setting;
2. The study type, classified as either a feasibility study or a diagnostic accuracy study;
3. The US transducer and frequencies utilized;
4. Achieved spatial resolution;
5. The method of tracking or image registration;
6. Computer applications or software used for reconstruction;
7. Reported accuracy measurements;
8. The time necessary for scanning procedure;
9. The samples used for scanning analysis;
10. The investigated potential applications for use in dentistry.

Studies were classified as feasibility studies when focused on technical implementation and workflow integration, and as diagnostic accuracy studies when quantitative comparison to a reference standard was performed. Given the absence of standardized classification systems, methodological categories were developed iteratively using an inductive approach during data extraction.

Reconstruction approaches were categorized as:

- Mechanized linear-stage raster scanning;
- Robotic arm-controlled multi- degree of freedom (DOF) scanning;
- Freehand scanning (tracked or untracked);
- Custom intraoral fixtures or positioning jigs.

Dental applications were categorized as:

- Anatomical structure visualization;
- Digital impressions and prosthodontics;
- Periodontal diagnosis and implant planning.

### Quality appraisal and reporting framework

To ensure methodological rigor and transparency across in vivo and in vitro studies, we employed a multi-tiered evaluation strategy. The main framework for the customized approach was the QUADAS-2 [20] tool, which is specifically designed for diagnostic accuracy studies. However, some aspects of the studies that focused on laboratory-specific sources of potential bias, such as specimen preparation, equipment calibration, positioning, coupling conditions, and reproducibility, were not adequately covered by this approach. Therefore, selected elements from the QUIN [21] framework were incorporated to address these concerns relevant to in vitro research. Finally, selected elements of the STARD [22] checklist were added for an evaluation of sample size justification, reporting of precision/uncertainty, and whether a predefined protocol or methodology was reported.

The customized, hybrid approach intention was to use QUADAS-2 as the diagnostic-accuracy foundation, QUIN to capture laboratory specific sources of bias, and STARD elements to improve assessment of methodological transparency and statistical reporting. Items were selected if they were relevant to our specific study and design.

This allowed for domain-level judgments (Low, High, or Unclear) for 14 checkpoints across 4 domains: sample selection, index test, reference standard and experimental setup.

The overall risk of bias was assessed as high if at least one criterion was high, and low if all categories were low. Otherwise, the risk was rated as unclear.

The customized criteria for the risk of bias and reporting guidelines created and evaluated for can be seen in Table S2.

## Results

Between five databases in the initial search, 850 articles were identified, with 237 being duplicate records. The second search identified an additional 51 articles. After screening by title and abstract, an additional 635 articles were removed. Full-text was retrieved and reviewed for 29 articles, with 3 being removed due to not being an experimental study, 14 not including a 3-dimensional reconstruction method and 3 not related to a dental clinical application. While reviewing the full text, an additional 4 articles were identified from reference checking. In total, 13 articles matched the inclusion and did not match the exclusion criteria and were included in the systematic review.

**Fig. 1:**
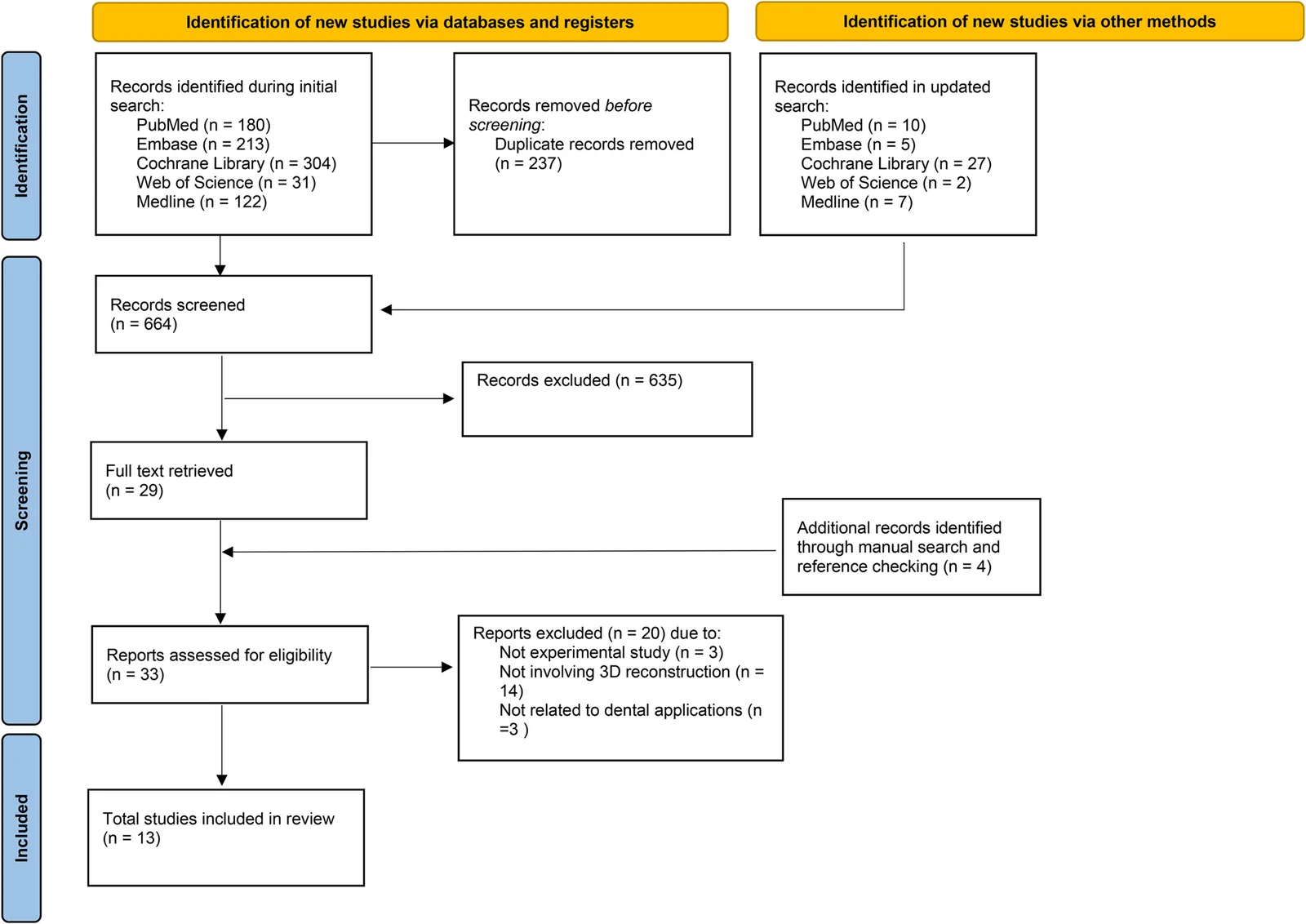
PRISMA flowchart

A PRISMA flowchart of the article selection process is displayed in Fig. 1.

The 13 included studies spanned from 2010 to 2025. Out of these, 10 (77%) were completed in an in vitro setting while 3 (23%) were conducted in vivo. The studies were classified as mainly a diagnostic accuracy study (5 studies) or mainly a feasibility study (8 studies). The US frequencies used ranged from 10 MHz all the way up to 150 MHz in High-Frequency (HFUS) applications.

The number of studies that utilized the techniques for US tracking included 6 mechanized linear-stage raster scanning, 2 studies with robotic arm-controlled multi-DOF, 2 studies with a tracked freehand US method, 1 study with untracked freehand scanning, and 2 studies with a custom intraoral jig/fixture.

For dental applications, three studies evaluated purposes related to visualization and evaluation of anatomical structures, five studies investigated utilizing 3D-US as a digital impression method, and five studies were used for purposes related to periodontal diagnosis and implant planning.

Table 1 highlights the extracted data from each article.

**Table 1:** Summary information from included studies

Author	Study Type	US System	Resolution	Tracking	Computer Applications	Accuracy	Performance Time	Test Samples	Applications
Mahmoud *et al.;* 2010 [23]	In Vitro Accuracy	Olympus NDT: 18–79 MHz	Axial: 24 μm Lateral: 123 μm	Two-axis positioning; controlled by PC	Control: Labview 8.2 Reconstruction: Matlab 7.1	Horizontal error: ±8.94% Vertical error: ±5.44%	Less than 30 s	Dry cadaver mandibles	Assessment of periodontal bony defects
Heger et al.; 2011 [29]	In Vitro Feasibility	HFUS Vevo 2100: 75 MHz	~ 30 × 30 μm	4-axis robot •3 translat-ional and 1 rotational DOF	Sparse Spatial Compounding for improved data coverage	Almost homogeneous distribution of digitized tooth surface points	N/A	2 extracted human molar teeth	3D digitization of tooth surfaces for CAD/CAM restorations
Heger et al.; 2013 [30]	In Vitro Feasibility	Focused HFUS: 50–150 MHz	•50 MHz: ~62 μm •100 MHz: ~34 μm •150 MHz: ~20 μm	4-axis robot •3 translat-ional and 1 rotational DOF	Optical reference scans processed using Dental Designer (3Shape) exported as STL files	Max deviation near preparation margin: *+/- 50* μm	N/A	3 model teeth and 1 extracted human molar	Digital impression of prepared teeth
Cheumbou Pekam et al.; 2015 [24]	In Vitro Feasibility	Single element focused transducer: 75 MHz	Lateral: ~40–60 μm Axial: ~18 μm	3-DOF kinematic extra-oral micro scanner	Thin-plate spline robust point matching algorithm for segmentation and surface reconstruction	Mean discrepancy vs. optical scans: 14–53 μm (artificial teeth). Up to 53 μm avg. and 52 μm SD (human molar)	N/A	3 artificial teeth + 1 human molar with chamfer preparation	Digital impression taking of subgingival prepared teeth
Du et al.; 2017 [25]	In Vitro Accuracy	Single-element focused transducer P150-2, Olympus NDT: Center frequency 50 MHz	•Lateral: ~62.5 μm •Axial: ~18 μm	3D motorized translation device	Signal acquisition and processing (Visual C++) 3D volume reconstruction and visualization (VTK Visualization toolkit) RF data, corrected with frequency-dependent gain compensation	Compared with micro-CT, average measurement error 3.55%, mean enamel thickness difference: -1.5% to -5%	Data acquisition, signal processing, reconstruction and rendering: <15 min per tooth	4 extracted human permanent molars from subjects aged 20–30 s	3D reconstruction of enamel layer
Lin et al.; 2018 [33]	In Vitro Accuracy	Vevo LAZR Imaging System VisualSonics: 21–40 MHz	•US mode: 100 μm •Photo-acoustic mode: 300 μm	•Manual positioning •Volumetric scanning •Photoacoustic acquisition using alternating 680 and 800 nm excitation	ImageJ 1.48 for photoacoustic image analysis. Vevo LAB for 3D depth measurements on the sagittal view	•Bias values: Mesial: -0.18 mm, Buccal/Lingual: 0.08 mm, Distal: -0.21 mm •Gingival thickness bias: 0.07 mm	N/A	39 porcine teeth; 12 with artificially deep pockets	Noninvasive periodontal probing depth and gingival thickness measurement
Marotti et al.; 2018 [26]	In Vitro Feasibility	HFUS Olympus V3320 single-element transducer: 75 MHz	•Axial: ~18 μm •Lateral: ~60 μm	3D positioning unit with xyz movement	•MATLAB: Noise filtering, gingiva segmentation •CloudCompare: Point cloud meshing and 3D deviation analysis •ImageJ: Measurement of convergence angles	Mean deviation values: Optical vs. Optical: 12–15 μm Optical vs. US: 42–46 μm	Prototype scan: 5–6 h per tooth	10 extracted human teeth prepared with a chamfer finish line, covered with porcine gingiva (0.3–0.9 mm thick)	Digital impression of subgingival dental preparations
Moore et al.; 2018 [27]	In Vivo Accuracy	Laser-integrated HFUS; Visualsonics: 16–40 MHz	•US spatial resolution: 100 μm •Photo-acoustic mode: 300 μm	Stepper motor-controlled scanning	•Visualsonics software: Image acquisition and •ImageJ: Image analysis and profile measurements	Pocket depths measured with PA-US showed precision of 0.01 mm, relative standard deviation 10%	5 min per tooth	1 healthy human volunteer (22 F); anterior teeth	Non-invasive periodontal assessment
Bohner et al.; 2021 [34]	In Vitro Feasibility	HFUS prototype single-element transducer: 42 MHz	•Axial: 20 μm •Lateral: 70 μm	Stabilization tray with silicone for fixation over dental arch	•CloudCompare: 3D deviation analysis •Custom algorithms convert B-mode images into 3D point clouds	•Mean discrepancy: US = 57.4 μm; Lab scan = 75.4 μm; Intraoral scan = 38.6 μm	2 min per implant scan	Acrylic resin model with Straumann dental implant	Digital implant impression with US scanner
Chifor et al.; 2022 [32]	In Vivo Feasibility	3D HFUS linear transducer: 10–23 MHz	•2D US: 375 × 735 pixels; exact resolution not specified	Articulated measuring arm Evo 7 with spatial and temporal calibration	•Custom software: semi automatic annotation, 3D reconstruction •Python: AI model implementation •Mask R-CNN and U-Net: Deep learning models •OpenCV GUI: Image processing	•IoU ranged from 10% (perio pocket) to 75.6% (gingiva)	Prediction: •Mask R-CNN: <0.5s per image •U-Net: several predictions per second	52 teeth from 11 patients (20–68 years old) with periodontal health and periodontitis	Automatic segmentation of periodontal tissues for diagnosis and monitoring
Tattan et al.; 2022 [28]	In Vitro Accuracy	Clinical US scanner Mindray ZS3: 14 MHz	•Axial: 70 μm •Lateral: 70 μm •Elevational: 200 μm	•Probe attached to positioning arm controlled by MATLAB scripts •Custom frame with fiducial markers register facial and lingual scans	•MATLAB: Image acquisition, merging facial and lingual scans •3D Slicer: manual registration of US and CBCT volumes	•US vs. CBCT ICC: 0.890–0.988 •Mean differences: Range − 0.38 mm to + 0.07 mm	~ 20 s per side (facial or lingual)	7 porcine hemi-mandibles	3D imaging of the jawbone for ridge width determination
Alavi et al.; 2023 [31]	In Vitro Feasibility	US handheld intraoral linear transducer: Up to 23 MHz	•Voxel size: 0.1 × 0.1 × 0.1 mm •Tracking resolution: 0.2 mm	Optical tracking system with 4 infrared cameras (6 DOF poses)	•3D Slicer: reconstruction and visualization •MATLAB: pose regularization and algorithm implementation	•Absolute error: 0.08–0.24 mm •Relative error: 1.01–4.63% •Hausdorff distance: ~0.5 mm	N/A	Mandibular phantom	3D intraoral imaging of dento-periodontal structures
Nava et al.; 2025 [35]	In Vivo Feasibility	Mindray ZS3: 24 MHz	Axial: 32.1 μm	•Custom positioning device •Alignment through CAD-based positioning	•Blender: image alignment and implant planning •CoDiagnostIX: surgical guide design and deviation analysis	•US vs. CBCT error: angular = 5.27° ± 1.75°; apex = 1.41 ± 0.61 mm •US vs. implant position: angular = 2.63° ± 0.43°; apex = 1.26 ± 0.27 mm	Requires additional appointments	9 patients; 11 implants	US-guided implant planning and placement

Feasibility: Focus on technical validation and workflow improvement; Diagnostic Accuracy: focus on comparing ultrasound reconstruction to a gold standard

*US* Ultrasound, *HFUS* High frequency ultrasound, *DOF* Degrees of freedom, *CBCT* Cone beam computed tomography, *CAD* Computed assisted design, *CAM* Computed assisted milling, *IoU* Intersection over union, *MHz* Megahertz, *CEJ* Cemento enamel junction

The customized risk of bias and reporting guideline assessment resulted in mostly unclear to high risk of bias, with 6 articles rated as high risk of bias and 7 rated as unclear. The results of this customized risk of bias assessment with individual domain ratings is displayed in Table 2.

**Table 2:**
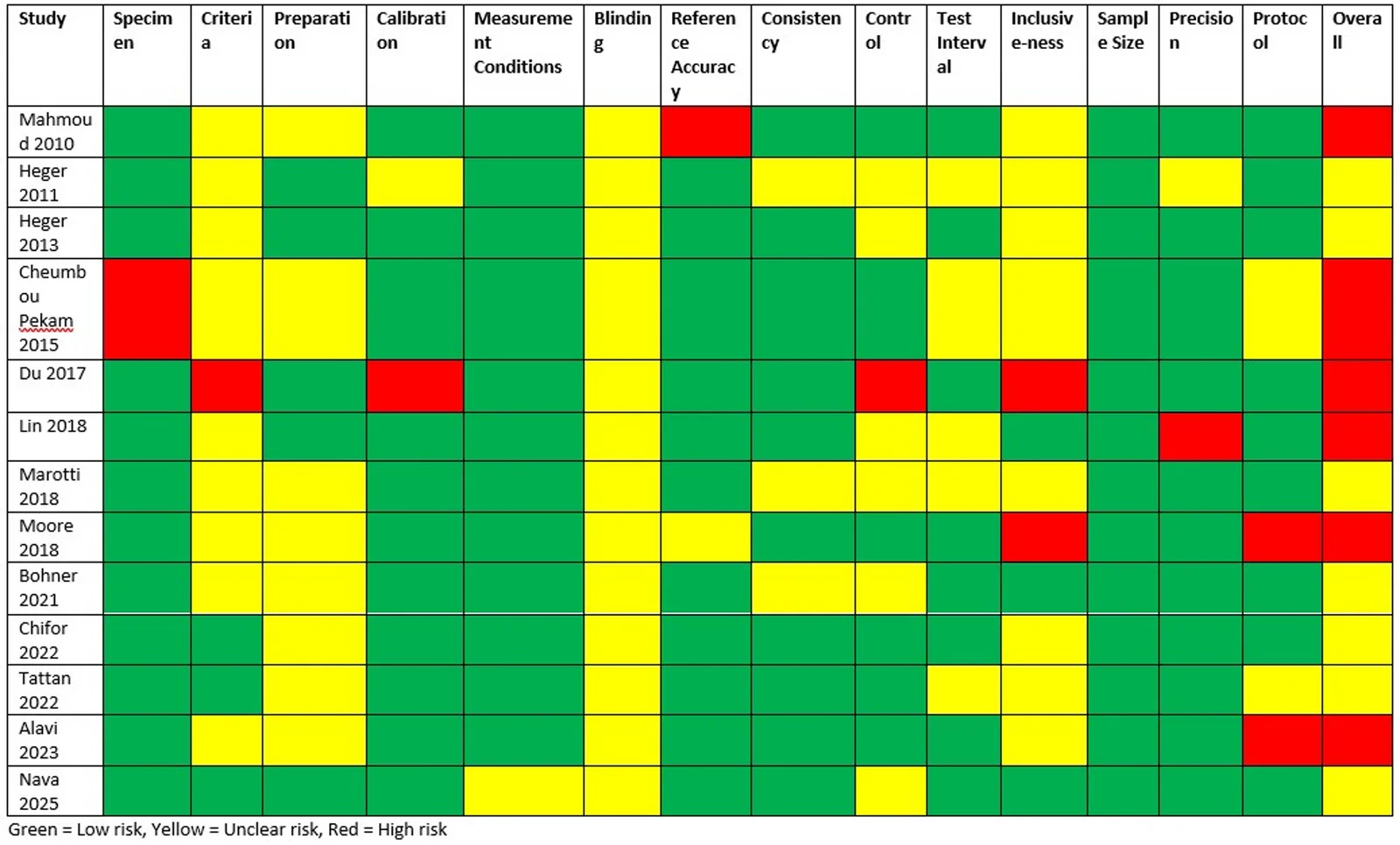
Custom QUADAS-2/QUIN and STARD risk of bias assessment

## Discussion

This study aimed to systematically collect and compare different methods that have been investigated for reconstruction of US images into a 3D model, as well as their potential applications and accuracy in dentistry. This process revealed studies as early as 2010 that have continued to progress until recently in 2025, with multiple methods of image tracking and reconstruction in both in vitro and in vivo environments. The analysis included cross-comparison of acquisition and reconstruction methods and analyzed the risk of bias through a hybrid framework.

### US tracking methods

The 13 studies were classified into various methods of tracking the 2-D US image for construction of a 3-D model.

#### Mechanized linear-stage scanning

Six studies (Mahmoud et al. [23], Chuembou Pekam et al. [24], Du et al. [25], Marotti et al. [26], Moore et al. [27], Tattan et al. [28]) employed a motorized translation stage to move the ultrasound transducer along predefined axes in a systematic raster pattern. The probe remained fixed in orientation while the stage executed controlled movements across X-Y and sometimes Z planes. A rigid frame with 2–3 DOF motorized linear stages provided movement increments in the sub-millimeter range. The scan pattern followed a raster pattern, sweeping across the specimen in parallel pattern layer by layer. In one of these studies (Tattan et al. [28]), separate scans were completed for the buccal and lingual aspects which were then merged using fiducial markers. The advantages of this technique were a true 3D reconstruction with uniform spatial sampling, high reproducibility and geometric accuracy without freehand variability. The reported limitations included the bulky and non-portable machine setup with relatively slower acquisition, limiting its use for clinical applications.

#### Robotic arm-controlled scanning

Two studies (Heger et al. (2011) [29], Heger et al. (2013) [30]) used an articulated robotic arm to position and move the ultrasound transducer. This technique enabled complex trajectories and multi-angle acquisitions and is designed for repeatable, automated scanning. The robotic arm contained 4 DOF integrated with a HFUS, which was then controlled via custom software for probe positioning and orientation. The probe orientation was able to be dynamically adjusted to maintain optimal incidence on curved surfaces. This allowed for advanced imaging modes such as harmonic imaging and spatial compounding. The reported advantages included high reproducibility and accuracy and automated multi-angle acquisition with reduced operator dependency. The limitations were due to the bulky and expensive robotic setup that required calibration and complex integration. Due to the setup, this technique would mainly be used for research setups.

#### Freehand scanning

There were 3 studies that utilized freehand or manual scanning. This method could be further divided into freehand scanning with a tracking method (Alavi et al. [31], Chifor et al. [32]) and freehand scanning without an additional tracking (Lin et al. [33]).

The tracked freehand scanning method combined the flexibility of freehand intraoral scanning with real-time pose tracking, enabling true 3D-US reconstruction. In the study by Alavi et al. [31], a handheld linear transducer was coupled with an optical tracking system of four infrared cameras that captured 6-DOF poses. Comparatively, the study by Chifor et al. [32] attached the US probe to an articulated measurement arm with spatial/temporal calibration which the operator could manipulate along the buccal and facial surface in a longitudinal orientation. The tracking methods utilized either infrared cameras or an attached articulating arm, but in both studies the US transducer was manually manipulated. The advantages of this technique included its portability and possibilities for clinical use while providing 3D imaging. The disadvantages included the necessity of maintaining line-of-sight for the optical tracking system and the added size of the articulating arm system, as well as the sensitivity to marker occlusion, calibration and operator skill.

Comparatively, the study by Lin et al. [33] employed manual intraoral US scanning without external pose tracking. In this study, photoacoustic-US imaging was obtained by oscillation between 680 and 800 nm wavelengths. The scanning collected a series of spatially separated image slices which were combined into a 3D volume through maximum intensity projection reconstruction. This untracked freehand scanning offered flexibility and chairside feasibility but lacked inherent spatial registration, limiting reconstruction applications and introducing operator-dependent variability.

#### Custom intraoral jig/fixture

Two studies designed custom fitted positioning devices. Bohner et al. [34] developed a partial tray covering 3–5 teeth to stabilize the US probe during scanning. This was then combined with mechanically driven two-DOF scanning for HFUS imaging. Nava et al. [35] used a 3D printed positioning guide based on intraoral scans and a digital wax up. The probe was limited to a prosthetically driven cross-sectional plane perpendicular to the arch. In both of these studies, there was improved reproducibility and reduced operator variability and better integration into digital workflows. However, they also introduced increased complexity for the design and fabrication steps of the fixture.

**Fig. 2:**
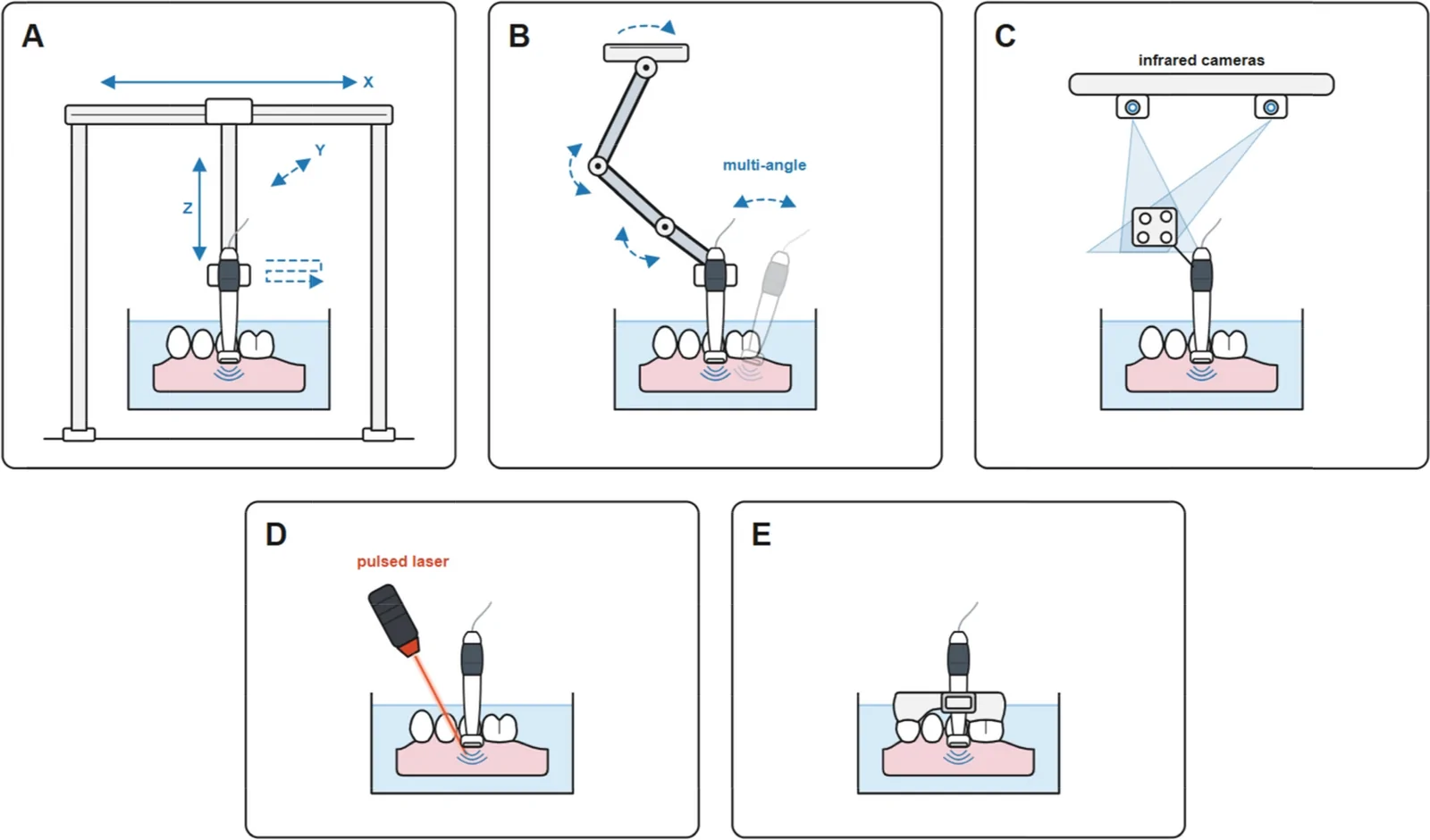
Illustrative diagrams displaying the various tracking methods. **A**) – Mechanized linear stage scanning. The transducer is attached to a motorized translation stage to move the ultrasound transducer along predefined axes in a systematic raster pattern. These were scanned linearly along one surface of the bone except for the study by Tattan et al. which scanned the buccal and lingual surfaces separately and then merged using fiducial markers; (**B**) – Robotic arm-controlled scanning. An articulated robotic arm was used to position and move the ultrasound transducer; (**C**) – Tracked freehand scanning. A handheld transducer was coupled with an optical tracking system of four infrared cameras that were able to capture positional and dimensional positioning of the transducer in space; (**D**) – Untracked freehand scanning. Alternating wavelengths of sound and light create a photoacoustic image that is merged for the resulting 3D image; (**E**) – Custom intraoral jigs or fixtures. A partial tray covering 3–5 teeth stabilized a US probe, which was either mechanically driven or manually operated

Example images illustrating the techniques described are displayed in Fig. 2. The images were generated by Claude Opus 4.8 (Anthropic, California, USA) and are meant as illustrative representations of methods described rather than technical diagrams.

### Summary of reconstruction methods

Each of the investigated methods presented displayed unique approaches to 3D-US reconstruction. Direct quantitative comparison across studies was not appropriate due to heterogeneity in reference standards, measurement endpoints, and reporting metrics. Comparatively, the reported accuracy levels for all systems remained relatively similar. If reported, the error rates ranged from 4.6 to 8.9%, while Nava et al. had error rates for implant placement of 2.6–5.2°. When comparing the volume reconstructed US images to other modalities, the differences were mostly reported in the µm range.

The robotic and mechanized approaches would be expected to have the highest reproducibility but likely represent the weakest clinical feasibility due to the size of the machined setup. Alternatively, a freehand tracked method likely holds the best clinical promise as it is most similar to current US use and was able to achieve moderate accuracy. This could be completed untracked, which was the least accurate but had simpler design. These approaches introduce variability to the 3D reconstruction but better reflect real-world constraints. Custom jigs have a moderate level of feasibility and good reproducibility. Finally, photoacoustic methods hold promise as a hybrid modality.

Although direct comparison was not possible, a consistent trade-off emerged across the included studies: systems designed to maximize positional accuracy and reproducibility generally required greater hardware complexity and were less suitable for chairside implementation, whereas clinically feasible approaches accepted some loss of reconstruction precision in exchange for workflow simplicity and intraoral adaptability.

**Fig. 3:**
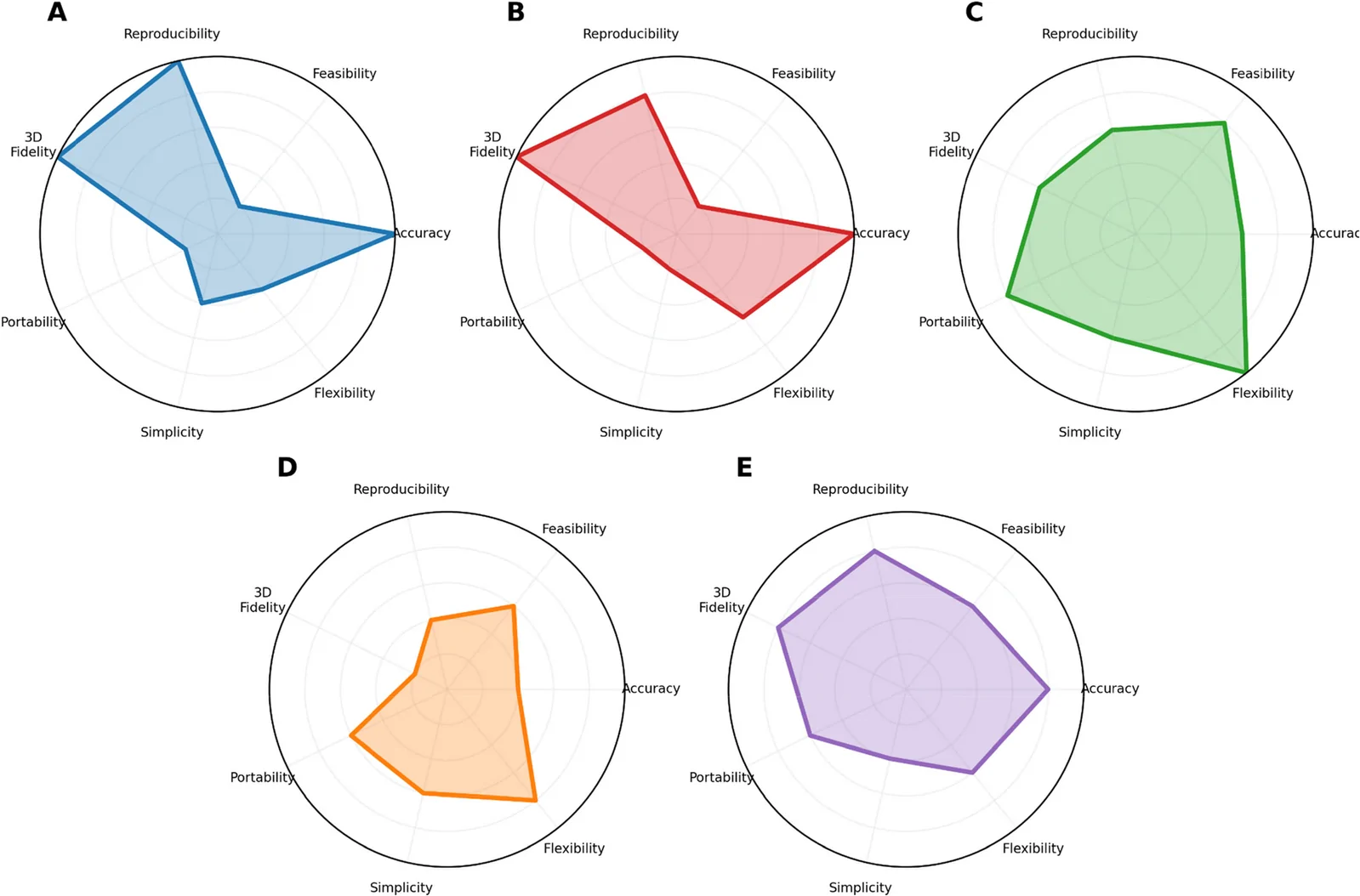
Radar charts for the various tracking methods highlighting the strengths and limitations of each technique. **A**) – Mechanized linear stage scanning; **B**) – Robotic arm-controlled scanning; **C**) – Tracked freehand scanning; **D**) – Untracked freehand scanning; **E**) – Custom intraoral jigs and fixtures

Table 3 provides a summary of the described the transducer tracking methods. Figure 3 provides an illustrative radar chart diagram highlighting the advantages and disadvantages of each technique.

**Table 3:** Summary of transducer tracking methods

Method	Accuracy	Clinical Feasibility	Pros	Cons
Mechanized Linear-Stage Scanning	•High •Sub-mm precision •Uniform sampling due to raster motion	•Low – requires bulky hardware with poor intraoral adaptability	•True 3D reconstruction with spatial control •High reproducibility with limited operator variability	•Non-portable, lab-based setup •Slow acquisition •Limited clinical use
Robotic Arm-Controlled Scanning	•Very high •Multi-angle, repeatable trajectories	•Very low- large, complex, expensive	•Automated, multi-angle acquisition •Dynamic orientation •High reproducibility •Enables advanced imaging modes	•Bulky and costly •Requires calibration and complex system integration •Limited clinical use
Freehand Scanning- Tracked	•Moderate to high •Dependent on tracking accuracy	•Moderate to high •Handheld system compatible with chairside use but requires external tracking	•Most clinically realistic •Flexible probe use •True 3D reconstruction •Demonstrated in-vivo use	•Optical tracking requires line-of-sight •Potential marker occlusion •Added size from tracking arms or cameras •Operator dependant
Freehand Scanning- Untracked	•Low to moderate •No inherent spatial registration •Varies by technique	•High •Simplest, most portable	•Maximum flexibility and simplest hardware •Chairside use •Compatible with hybrid methods	•Typically lacks explicit spatial registration •Lower reconstruction fidelity •High degree of technique and operator dependency
Custom Intraoral Jig	•Moderate to High •Improved reproducibility by stabilizing probe position	•Moderate •Requires individualized fabrication but fits clinic environment	•Increased reproducibility and reduced operator variability •Good alignment with digital dentistry •Improves standardization of probe placement	•Fabrication time and complexity •Limited to specific arches/tooth regions •Reduced flexibility compared to freehand use

### Dental applications

#### Anatomical visualization

Three studies described utilizing 3D-US techniques for visualization of anatomical structures. In two of these studies (Chifor et al. [32], Alavi et al. [31]), the dento-periodontal structures were evaluated, such as the cementoenamel junction, alveolar bone, gingiva, crown, root and periodontal pocket. From the 3D reconstructed volume, automatic segmentation and integrated machine learning were implemented for feature detection. One study (Du et al. [25]) used the 3D-US image to evaluate the enamel thickness for detection of erosion or dental caries. The evaluation for enamel erosion and caries was able to achieve relatively greater spatial resolution in comparison to the studies evaluating for dento-periodontal structures.

#### Digital impressions

There were five studies (Heger et al. (2011) [29], Heger et al. (2013) [30], Chuembou Pekam et al. [24], Bohner et al. [34] and Marotti et al. [26]) that included 3D-US images for digital impressions. Traditional and intraoral camera impression techniques may have difficulty in imaging past gingival tissue. US imaging has been explored as an alternative that is capable of imaging the tooth or implant margin relative to the bone when it is covered by gingival soft tissue. HFUS images were used in all five studies to create a digital impression for crowns, CAD/CAM restorations, for dental implants and for evaluation of a subgingival margin detection.

#### Periodontal diagnosis and implant planning

There were five studies (Moore et al. [27], Lin et al. [33], Tattan et al. [28], Mahmoud et al. [23], Nava et al. [35]) categorized under periodontal diagnosis and implant planning. The jawbone surface was able to be visualized with a 3D-model and osseous defects could be seen. A 3D assessment evaluated the overall ridge width, which then could use US for planning and guiding dental implant placement. In the presence of periodontal pocket formation, the 3D model could visualize the structure of the pocket and could measure the pocket depth in a pain-free method compared to traditional periodontal probing.

#### Accuracy and scanning time

Across studies, the accuracy metrics reported indicated millimeter to submillimeter accuracy levels compared to gold standards and error rates that did not exceed 10%. The 3D-US systems reported accurate preparation margins for restoration impressions, periodontal pocket assessments that may be more accurate than traditional methods, with mostly accurate representations of relevant anatomy and the system showed promise to aid in implant planning assessment and guided surgery. However, in the visualization of subgingival margins, the 3D-US method displayed greater standard deviations compared to optical scanners while in implant surgery, the 3D-US guided method display greater degree of error compared to CBCT.

#### Clinical translation barriers

The findings of this review are broadly consistent with previous systematic reviews of dental ultrasonography, which identified methodological heterogeneity, lack of dedicated intraoral hardware, operator dependency, and limited integration into digital workflows as barriers to adoption. While those reviews evaluated two-dimensional US applications, the current review demonstrates that many of the same challenges remain present within 3D-US systems and may be amplified by the additional requirements for spatial tracking, 3D reconstruction, and registration. Consequently, standardization of acquisition protocols, reconstruction workflows, validation methods, and accuracy-reporting metrics remains an important need for future investigation.

The articles identified in this systematic search ranged from 2010 to 2025, the earliest in vivo use was in 2018. Among the 13 identified studies, the majority (10) were performed in vitro, and most (8) were categorized as feasibility studies centered on technical implementation rather than diagnostic accuracy. While these techniques could provide significant advantages for visualization of various structures, the low number of in vivo and accuracy studies likely illustrates the difficulty in translating these techniques to clinical use.

From the studies classified as mainly feasibility studies, the scanning time ranged from 2 minutes up to 5–6 hours, and it was clarified that the 3D-US setup may also require additional clinical appointments for the patient. The studies that were mainly classified as diagnostic accuracy studies comparatively had scanning times from less than 30 s per tooth up to 15 minutes.

In current practices, these systems require larger scanning setups or platforms, possibily including machine or robotic attachments, high powered computing, and increased scanning and acquisition times. Given the small oral environment, the increased equipment sizes may pose significant challenges for dental applications. Despite the added information from the volumetric collection of data, the images are still subject to other limitations of US imaging, such as the need for coupling agents, the inability to visualize past cortical bone and being operator dependent in freehand methods or inaccessible for clinical use in machined methods.

These implementation challenges are not uniform across methods. Mechanized linear-stage and robotic arm-controlled systems currently remain primarily research-based technologies due to their physical footprint, cost, calibration requirements, and limited intraoral adaptability. In contrast, tracked freehand systems demonstrated the strongest potential for chairside implementation because they employ handheld transducers and have already been evaluated in clinical settings; however, additional tracking methods introduce greater complexity compared to traditional US scanning. Custom intraoral guide systems may represent an intermediate approach, offering improved reproducibility while remaining compatible with digital dentistry workflows, although additional fabrication and planning steps are required.

One of the frequently listed limitations of US in dentistry is operator dependence and inter-operator reproducibility. For the proposed 3D-US methods, the mechanized and robotic approaches inherently minimized the operator-related variation, but at the expense of requiring larger setups with limited practicality for clinical settings. The freehand methods, whether tracked or untracked, remained dependent on consistent transducer placement and accuracy and therefore would retain the same limitations of operator dependence. The limited number of in vivo investigations and absence of formal reproducibility studies currently make it difficult to determine how reliably these systems would perform across operators with varying levels of US experience.

None of the included studies included descriptions of cost-effectiveness. However, the additional hardware requirements would be expected to introduce another barrier for clinical introduction due to the economic requirements.

In addition to these barriers, the currently investigated applications remain focused primarily on dental hard tissues such as teeth and periodontal/peri-implant bone. Our search strategy specifically identified studies relevant to dental or dentistry likely influenced these results. US has proven useful in multiple related areas of the orofacial environment, such as evaluation of the muscles of mastication, salivary glands, tongue, or lymph nodes. 3D reconstruction of US images may prove useful in these midfacial regions that are close in location and related in function to the dental environment and may have been underrepresented by our review. However, the nature of soft tissues being more susceptible to movement could create an additional challenge to the methods described above.

Several studies proposed 3D-US as a complementary technology to optical intraoral scanning. Unlike optical scanners, US can visualize structures beneath the gingival surface, including preparation margins, periodontal tissues, and peri-implant structures. However, the current methods required greater acquisition and reconstruction complexity and generally demonstrated slower acquisition times than optical scanners. Future work could incorporate multimodal systems combining optical surface

Table 4: Summary of the dental applications of described 3D-US methods.

**Table 4:** Summary of the dental applications of described 3D-US methods

Application	Representative Studies	Primary Capabilities	Reported Accuracy	Clinical Feasibility	Key Limitations
Anatomical Visualization	Chifor, Alavi, Du	•CEJ detection •Gingiva, alveolar bone, pocket visualization •Enamel thickness	•Sub-mm accuracy in enamel and bone measurements •CEJ localization error ~ 0.23 mm	•Moderate	•Soft tissue motion artifacts •Limited US penetration •Operator dependent
Digital Impressions	Heger (2011), Heger (2013), Cheumbou Pekam, Bohner, Marotti	•HFUS-based digital impressions for crowns, restorations, subgingival margin detection	•12–50 μm Deviation vs. optical scans •Subgingival margin detection less precise than optical scanners	•Low- primarily in vitro mechanized/ robotic systems	•Bulky systems •Long scanning times •Limited intraoral access
Periodontal Diagnosis and Ridge/Implant Planning	Moore, Lin, Tattan, Mahmoud, Nava	•Ridge width measurement •Jawbone and osseous defect visualization •Pocket depth measurement •Implant planning/ guidance	•~0.01–0.3 mm periodontal pocket precision •Implant navigation errors ~ 1–1.4 mm	•Moderate •In vivo freehand and custom guide systems demonstrated •Implant planning feasible	•Requires stabilization or guidance systems •Reduced accuracy vs. CBCT •Limited bone visualization

### Risk of bias assessment

The customized risk-of-bias assessment found that all included studies were judged as having either unclear or high risk of bias. These findings should be considered when interpreting the reported performance metrics, as many studies were exploratory feasibility investigations with small sample sizes, limited reporting of methodological details, and heterogenous reference standards. Quantitative synthesis was not undertaken because the included studies also exhibited substantial methodological heterogeneity. The evidence included both in vitro and in vivo investigations with objectives ranging from technical feasibility to diagnostic accuracy. Furthermore, the studies utilized different US systems, acquisition workflows, reconstruction methods, reference standards, and outcome measures, making statistical pooling unlikely to generate meaningful or reliable summary estimates. Consequently, the available evidence should be viewed primarily as demonstrating technical feasibility and development rather than providing definitive evidence of clinical effectiveness or readiness for widespread clinical implementation.

### Future developments

Future developments in this field may continue to investigate additional methods for creating a 3D-US reconstruction with a method that has higher simplicity and a decreased physical footprint. Several factors could contribute to this development. Some of these factors may include:

- Accessibility to high-power computing applications;
- Tracking methods without line-of-sight constraints;
- Merging of multiple image modalities into unified workflows;
- Artificial Intelligence-assisted automation and reconstruction.

These possibilities, together with the advancements seen over this review’s timeframe, point toward potential future clinical applications, although substantial validation and workflow challenges remain. Future systems are expected to emphasize smaller transducers and scanning hardware which will enhance intraoral manuevering and chairside use. Improvements in spatial localization may overcome current limitations associated with optical tracking or bulky mechanical fixtures. In addition, deep-learning-based reconstruction is likely to reduce operator dependency and shorten reconstruction time.

In addition, incorporation of multi-modal fusion, such as aligning US data with optical scans or low-dose CBCT, may enhance the anatomical structures visualized. As these technologies mature, standardized validation frameworks and clinically oriented accuracy studies will be essential to ensure reproducibility and integration into digital dentistry ecosystems. Currently, only 3 of the studies identified were performed in vivo, which highlights the current challenges of introducing these techniques for clinical practice. However, the advancements collectively demonstrate that 3D-US possesses promising technical feasibility and preliminary evidence supporting future clinical translation. While the current evidence remains predominantly preclinical, further refinement of workflows, tracking systems, and reconstruction methods may allow 3D-US to emerge as a valuable radiation-free modality for diagnostic imaging in dentistry.

## Conclusion

This systematic review demonstrates that 3D-USin dentistry has advanced over the past decade, with diverse acquisition, tracking, and reconstruction approaches enabling increasingly accurate visualization of dento-periodontal structures. Mechanized and robotic systems continue to offer the highest geometric fidelity, while tracked and untracked freehand approaches appear to offer the strongest pathway toward future clinical integration. However, most studies remain feasibility-based and in vitro, and significant variation in methodology, reporting, and reference standards limits comparability and clinical translation. Continued progress will depend on standardized validation frameworks, increased in vivo accuracy studies, and further automation of acquisition workflows. While the current evidence remains predominantly preclinical, the potential ability to generate 3D diagnostic information in a dental clinical setting without exposure to ionizing radiation continues to justify further development and clinical evaluation of 3D-US technologies in dentistry.

## Supplementary Information

Below is the link to the electronic supplementary material.

Supplementary Material 1 (DOCX 17.2 KB)

## Data Availability

No datasets were generated or analysed during the current study.
