# Supplementary material for: Three-dimensional reconstruction of ultrasound imaging in dentistry: a systematic review
Source: Clin Oral Investig. 2026 Nov 5;30(11):553. doi: 10.1007/s00784-026-07243-6 (PMC13736472; doi:10.1007/s00784-026-07243-6)
Supplement: Supplementary file 1 [file 784_2026_7243_MOESM1_ESM.docx]

**Supplementary Materials**

**Supplementary Table S1:** Specific search terms used for all databases

| **Search June 2025** | **Search Strategy** |
| --- | --- |
| PubMed  Before 2025 -02-28 (search 1)  Before 2025-12-31 (search 2)  (Record number = 180) | ((Ultrasound[Title/Abstract]) OR (US[Title/Abstract]) OR (Ultrasonography[Title/Abstract])) AND ((3D[Title/Abstract]) OR ("3 dimensional"[Title/Abstract]) OR ("three dimensional"[Title/Abstract])) AND ((Dentistry[Title/Abstract]) OR (Dental[Title/Abstract])) |
| Embase  Before 2025-02-28 (Search 1)  From 2025/2/28 to 2025/12/31 (Search 2)  (Record number = 213) | ((Ultrasound:ti,ab) OR (US:ti,ab) OR (Ultrasonography:ti,ab))  AND ((3D:ti,ab) OR ("3 dimensional":ti,ab) OR ("three dimensional":ti,ab))  AND ((Dentistry:ti,ab) OR (Dental:ti,ab)) AND [01-01-1000]/sd NOT [01-03-2025]/sd |
| Cochrane Library  Before 2025-02-28  From 2025/2/28 to 2025/12/31 (Search 2)  (Record number = 304)  (Word variations have been searched) | #1 ((Ultrasound:ti,ab) OR (US:ti,ab) OR (Ultrasonography:ti,ab)) AND ((3D:ti,ab) OR ("3 dimensional":ti,ab) OR ("three dimensional":ti,ab)) AND ((Dentistry:ti,ab) OR (Dental:ti,ab))  #2 (dental):ti,ab,kw AND (ultrasound):ti,ab,kw |
| Web of Science  Before 2025  Limited to 2025 (Search 2)  (Record number = 31) | ((Ultrasound or US or Ultrasonography) (Abstract) and (3D or "3 dimensional" or "three dimensional") (Abstract) and (Dentistry or Dental)) (Abstract) |
| Medline  Before 3/2025 (Search 1)  From 3/2025 to 12/2025 (Search 2)  (Record number = 122) | ((Ultrasound or US or Ultrasonography) and (3D or "3 dimensional" or "three dimensional") and (Dentistry or Dental)) |

**Supplementary Table S2:** Customized criteria and guidelines for risk of bias assessment and reporting quality across in vitro and in vivo feasibility and accuracy studies. All signaling questions were rated as either Low, High or Unclear.

| **Domain** | | **Signaling Question** | **Source** | **Adaptation Note** |
| --- | --- | --- | --- | --- |
| **1. Sample Selection** | 1a. Specimen Type | Was the type of specimen (tooth/bone/model) appropriate and representative for the intended diagnostic application? | QUADAS-2 | Replaces patient selection with specimen/model selection. |
|  | 1b. Selection Criteria | Were inclusion and exclusion criteria for the specimens clearly defined and justified? | QUIN | Derived from QUIN’s clarity of eligibility criteria. |
|  | 1c. Specimen Preparation | Was specimen preparation standardized (cleaning, mounting, hydration, defect creation)? | QUIN | Relevant for reproducibility in lab-based studies. |
| **2. Index Test** | 2a. Device Calibration | Was the ultrasound device (transducer, robot, positioning system) calibrated or validated prior to testing? | QUIN | Reflects equipment/operator calibration needs. |
|  | 2b. Measurement Conditions | Were measurement conditions standardized (coupling medium, stabilization, alignment, environment)? | QUIN | Laboratory-specific condition control; no QUADAS-2 equivalent. |
|  | 2c. Interpretation Independence | Were the index test results interpreted independently of the reference standard? | QUADAS-2 | Directly taken from the original domain. |
| **3. Reference Standard** | 3a. Reference Accuracy | Is the reference standard (micro-CT, CBCT, optical scanner, periodontal probe) likely to provide accurate classification/measurement? | QUIN | Directly taken from the original domain. |
|  | 3b. Reference Consistency | Was the reference standard applied consistently and appropriately across all specimens? | QUIN | From QUIN’s reproducibility/consistency criteria. |
| **4. Experimental Setup/Flow & Timing** | 4a. Experimental Control | Were the experimental conditions adequately controlled (robot/arm precision, positioning repeatability, software consistency, hydration, temperature)? | QUIN | Domain created for lab-based bias sources |
|  | 4b. Test Interval | Was the interval between the index test and reference standard negligible or standardized? | QUADAS-2 | Retains QUADAS-2 flow/timing but adjusted for ex vivo sequencing. |
|  | 4c. Sample Inclusion | Were all specimens included in the final analysis, or were exclusions justified? | QUADAS-2 | Standard QUADAS-2 assessment of attrition. |
| **5. Reporting and Statistical Validity** | 5a. Sample Size Justification | Was a sample size justification or rationale provided? | STARD | Added to address methodological transparency in laboratory validation studies. |
|  | 5b. Precision Reporting | Were measures of precision or uncertainty reported (e.g., SD, CI, reproducibility metrics, ICC)? | STARD | Ensures statistical robustness and reproducibility reporting. |
|  | 5c. Protocol Transparency | as a study protocol, preregistration, or predefined methodology reported? | STARD | Improves transparency and reduces selective reporting bias. |
